# Ultrasound-guided block of the superior cervical ganglion for migraine attacks: a propensity score-matched retrospective study

**DOI:** 10.3389/fpain.2025.1556654

**Published:** 2025-08-28

**Authors:** Wenxing Zhao, Hong Yue, Liqiang Yang, Liangliang He

**Affiliations:** ^1^ Department of Pain Management, Xuanwu Hospital, Capital Medical University, Beijing, China; ^2^ Department of Pain Management, Peking University Shougang Hospital, Beijing, China

**Keywords:** migraine attack, superior cervical ganglion block, ultrasound, monthly migraine days, migraine disability

## Abstract

**Background:**

This study aimed to examine the efficacy and safety of ultrasound (US)-guided superior cervical ganglion (SCG) block in conjunction with standard triptan in the management of migraine attacks.

**Methods:**

In total, 243 subjects who received an adjunctive US-guided SCG block alongside triptan for a migraine attack were enrolled as the SCG cohort. A 1:1 propensity score based on baseline covariates was used to match 243 cases who received triptan alone as the control. The primary endpoints were pain relief and freedom from pain within 24 h after the procedure. Secondary outcomes included headache relief and freedom from pain within 2 h, monthly migraine days (MMDs), Migraine Disability Assessment (MIDAS) scores, Migraine-Specific Quality of Life questionnaire (MSQ) scores, and adverse events.

**Results:**

The rates of pain relief and freedom from pain at 24 h after the block were increased in the SCG cases compared to the controls {73.3% vs. 49.4%, with mean difference [MD] of 23.9% [95% confidence interval (CI): 15.5%–29.0%] and 64.2% vs. 37.4%, with MD = 26.7% [95% CI: 18.2%–31.3%], respectively}. Superiority was met, as the 95% CI fell within the superiority margin of 15%. Higher rates of pain relief and freedom from pain at 2 h following the procedure were reported in the SCG cohort (both *p* < 0.001). At the 1-month follow-up, the SCG cohort had a greater improvement in MMDs (*p* < 0.01), MIDAS scores (*p* = 0.040), and MSQ scores (*p* = 0.036). There were no severe adverse events in the SCG group.

**Conclusions:**

US-guided SCG block with triptan was superior to triptan alone in achieving headache remission during a migraine attack for up to 24 h, resulting in reduced migraine days and improved functional ability and life quality at the 1-month follow-up.

## Introduction

Migraine is a common neurological disorder characterized by episodes of disabling headaches (1). Its prevalence increased to 7.0% among the Chinese population between 2000 and 2023 (2). A migraine poses a serious impairment to a patient’s quality of life and social activities, placing an enormous burden on the healthcare system with the age-standardized disability-adjusted life years (DALYs) of 422 cases per 100,000 people in the Asia-Pacific region (3). Although the physiopathology of migraine is complex, peripheral structures innervated by the trigeminocervical complex and by the upper cervical spine may generate and maintain nociceptive afferents, which, in turn, may be responsible for peripheral sensitization. As a consequence, peripheral sensitization could lead to an increase in the stimulation of the brain stem, hypothalamus, thalamus, and cortex, which, in turn, could lead to central sensitization (4, 5). Acute treatment aims to stop the progression of migraine attacks, with oral non-steroid anti-inflammatory drugs (NSAIDs) and triptans recommended as first-line options based on the International Headache Society guidelines (6). Recently, in patients whose migraines persist despite medications, a stellate ganglion block (SGB) can be used early as an adjunctive treatment for symptomatic relief of acute migraines (7). Given that the sympathetic fibers modulating the vasomotor function of the meningeal artery and dural sinuses mainly originate from the largest cervical sympathetic ganglion of the superior cervical ganglion (SCG), SCG blockade, instead of SGB, may be promising in the management of migraine (8, 9).

Although the utility of SCG blockade has been reported for headaches or orofacial pain in some cases, evidence for migraine is limited (10). We hypothesized that blocking the cervical sympathetic ganglion might interrupt the nociceptive signals that activate the aforementioned trigeminal-autonomic reflex, thereby terminating the migraine attack. Consequently, this study aimed to evaluate the efficacy and safety of ultrasound (US)-guided SCG block with fluoroscopic (FL) verification as an adjunctive treatment to standard triptan for the treatment of a migraine attack in comparison to triptan alone.

## Methods

### Study design and subjects

This retrospective cohort study was approved by the institutional Ethics Examining Committee of Human Research (XW-KY-202417) according to the principles of the Declaration of Helsinki, following the Strengthening the Reporting of Observational Studies in Epidemiology (STROBE) guidelines (11). Written informed consent was waived because all data were retrieved from medical records.

The records of subjects admitted to our pain clinic for the treatment of migraine attacks between 1 January 2024 and 31 January 2025 were extracted from the electronic medical records (EMRs). Inclusion criteria were as follows: (1) a diagnosis of migraine per the International Classification of Headache Disorders (ICHD), 3rd edition (12); (2) a history of migraine for at least 12 months; (3) an untreated migraine attack lasting 4–72 h; (4) moderate or severe pain intensity; (5) aged ≥18 years old. Subjects who had received prophylactic pharmacological and non-pharmacological treatments were excluded, and further exclusion criteria included utilization of triptans within the prior 72 h, another headache due to organic disorders, menstrual migraine, severe systematic dysfunction, confounding chronic pain requiring regular analgesia, coagulation disorders, allergy to contrast medium, psychiatric illness, pregnancy/lactation, lost to follow-up due to converting to other procedures, or incomplete data.

**Figure 1:**
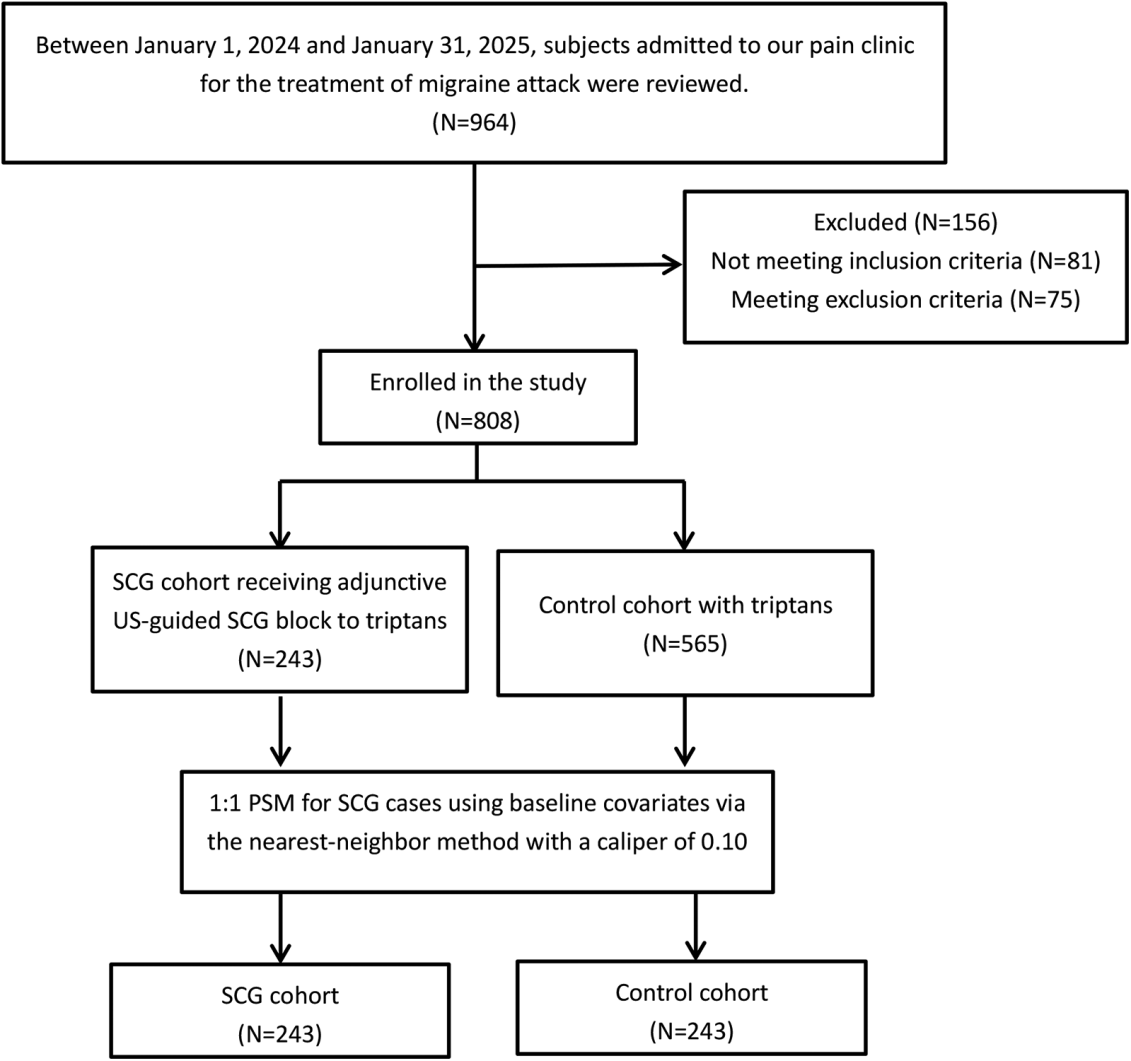
The flow chart of the study cohorts. SCG, superior cervical ganglion; US, ultrasound; PSM, propensity score match.

Thus, 243 consecutive subjects who received a US-guided SCG blockade were identified as the SCG cohort. Given that both SCG blockade and standard triptans were routine treatments for a migraine attack, subjects were requested to select the management option that most closely aligned with their values and preferences after consulting with their doctor. Therefore, propensity score matching was performed in subjects who received standard triptans alone in a 1:1 ratio to create a control cohort (Figure 1). The score was estimated using the subjects' baseline covariates via the nearest-neighbor method with a range of 0.10 as follows: (1) age, (2) gender, (3) body mass index (BMI), (4) diagnosis type, (5) affected side, (6) migraine attack duration, (7) medication type, (8) prior preventive treatment attempts, (9) headache severity, (10) comorbidities, and (11) triptan dosage. According to the Chinese guidelines for the treatment of a migraine attack, zolmitriptan tablets were provided at a dosage of 2.5 mg, and a second dose was available if symptoms persisted after 2 h. The maximum dose should not exceed 15 mg within 24 h (13). Celecoxib tablets (200 mg, up to two times daily) was permitted as rescue analgesia if the patient’s visual analog scale (VAS) score was ≥4, while oxycodone and acetaminophen (5 mg: 325 mg tablets, up to four times daily) was available when the patient’s VAS score was ≥7 based on the recommendations of the analgesic ladder from the World Health Organization (WHO) (14).

### SCG block procedure

US-guided SCG blocks were performed by a team of pain doctors with a minimum of 5 years of experience using the minimally invasive techniques and US imaging for the management of headache.

**Figure 2:**
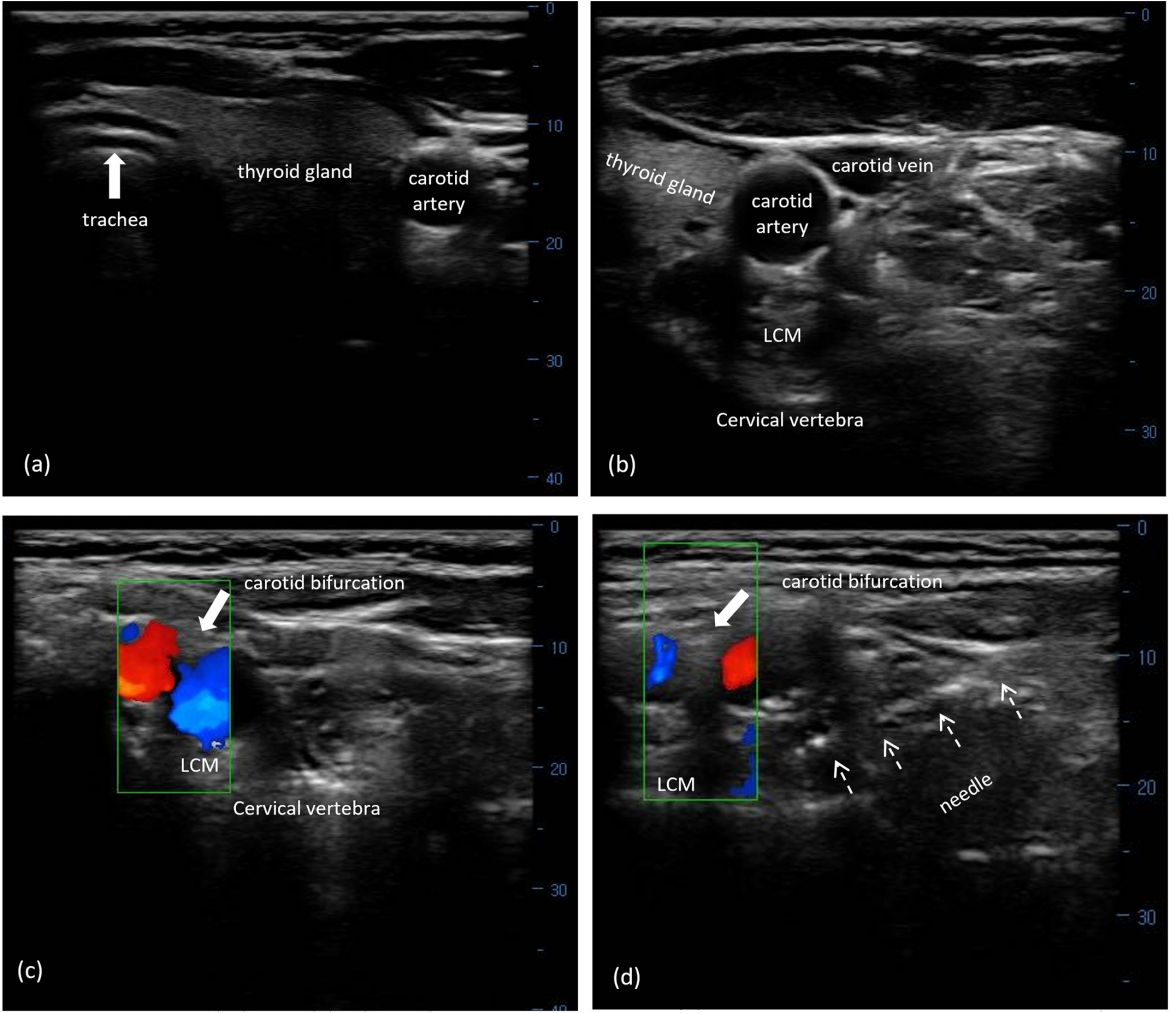
US-guided SCG block with FL verification. (**a**) Midline structures of the cervical region, including trachea, esophagus, and thyroid gland, on transverse US imaging. (**b**) The right carotid sheath and its surrounding structures in the lower cervical area. (**c**) The carotid bifurcation on the surface of the LCM using the Doppler model. (**d**) A needle (white arrows) was visualized from the lateral direction toward the targeted SCG between the carotid bifurcation and the LCM at the second to third cervical segment. US, ultrasound; SCG, superior cervical ganglion; LCM, longus capitis muscle.

The subjects were placed in a supine position with their neck slightly extended in the operating room. A linear 6–13 MHz probe was placed transversely over the ipsilateral side of their lower neck with an orientation to the midline. The trachea, esophagus, and thyroid gland were visualized when the probe was moved up or down (Figure 2a). The carotid sheath lying between the longus colli muscle (LCM) and sternocleidomastoid was subsequently identified by further laterally moving the probe (Figure 2b). The color Doppler mode was used to confirm the contrasting blood flow in the jugular vein and carotid artery. The carotid artery was identified by visualizing it as a pulsatile structure using B-mode in the US. Once identified, the probe was gradually advanced in the cephalic direction along the carotid artery until the carotid bifurcation was obtained (Figure 2c). The targeted SCG was located just posterior to the carotid bifurcation and anterior to the LCM in front of the second to third cervical transverse process (TP). Using a 22-gauge needle, the targeted SCG was approached using an in-plane technique under real-time guidance from the skin entry point, which was 1 cm lateral to the probe (Figure 2d). After negative aspiration, 1 mL of 0.9% sodium saline was injected beneath the fascial plane for visualization of the correct position. Accordingly, subjects were treated with an injection of 2% lidocaine (50 mg) and 5 mg: 2 mg/mL betamethasone (0.5 mL) diluted with normal saline to 5 mL. Subjects were evaluated for signs of Horner's syndrome at 5 min after injection, and routinely monitored using electrocardiography, blood pressure, and oxyhemoglobin saturation every 5 min for a total of 20 min.

### Outcomes measurement

Demographic details and clinical characteristics were retrieved from the EMRs. Follow-up data collection was routinely performed at the time of enrollment in our pain clinic by the doctors in charge, and at intervals of 2 hours, 24 hours, and 1 month through telephone interviews by two specially trained nurses, and registered in our prospectively maintained database.

The pain severity of the migraine attack was assessed using an 11-point numeric rating scale (NRS), which ranged from 0 to 10 with 0 = no pain, 1 to 3 = mild, 4 to 6 = moderate, and 7 to 10 = severe (15). Pain relief from acute migraine was defined as the pain being reduced from moderate or severe severity to none or mild, while freedom from pain was defined as the headache being completely relieved from moderate or severe severity. Monthly migraine days (MMDs) were defined as the change in the number of days with a migraine over 1 month after treatment from baseline (16). The Migraine Disability Assessment (MIDAS) was utilized to estimate headache-related disability (17, 18). The Migraine-Specific Quality of Life questionnaire (MSQ) version 2.1 was used to measure the subjects' quality of life (QoL) (19, 20). Adverse events associated with oral triptan or SCG block were recorded.

The primary endpoints were pain relief and freedom from pain at 24 h after SCG block. A superiority margin of 15% was predefined for the subjects in the SCG cohort because headache relief of 50% at 24 h has been observed after oral triptan according to a systematic review and network meta-analysis (21). Secondary outcomes included pain relief and freedom from pain at 2 h following the procedure, MMDs, MIDAS scores, MSQ scores, rescue analgesics, and adverse events at the 1-month follow-up.

### Statistical analysis

SPSS software version 22.0 (SPSS Inc., Chicago, IL, USA) was used for the statistical analysis. Significance was defined as *p* < 0.05. All data were checked for normality using the Kolmogorov–Smirnov *Z* test. Normally distributed data were recorded as mean ± standard deviation (SD) and compared using Student’s *t*-test, while non-normally distributed data were expressed as median [interquartile range (IQR)] and compared using the Mann–Whitney *U* test. Categorical data were recorded as percentages and compared using the chi-squared test. The repeatedly measured data over time in the groups were compared via the repeated measures mixed-design analysis of variance (ANOVA), using their baseline values as covariates. *Post hoc* comparisons across the three time points within the groups were performed at an adjusted significance level of 0.05/3 = 0.017.

## Results

Figure 1 shows the flow diagram of the two cohorts. As shown in Table 1, no significant differences in baseline variables were observed between the two cohorts.

**Table 1:** Demographic and clinical characteristics of the participants at baseline.

Variable	SCG cohort	Control cohort	*P*-value
	(*N* = 243)	(*N* = 243)	
Age (years) (mean ± SD)	45.07 ± 7.51	46.03 ± 8.29	0.640
Gender, *n* (%)			
Female	152 (62.6%)	158 (65.0%)	0.321
Male	91 (37.4%)	85 (35.0%)	
BMI (kg/m^2^)	23.62 ± 2.53	24.58 ± 3.12	0.957
Diagnosis, *n* (%)			0.580
Migraine with aura	55 (22.6%)	49 (20.2%)	
Migraine without aura	188 (77.4%)	194 (79.8%)	
Duration of migraine (years)	12.04 ± 8.98	11.76 ± 6.64	0.939
Affected side, *n* (%)			0.920
Left	101 (41.6%)	97 (39.9%)	
Right	87 (35.8%)	88 (36.3%)	
Bilateral	55 (22.6%)	58 (23.9%)	
Headache attack duration (h)	93.84 ± 17.09	90.42 ± 12.02	0.467
Acute headache medication, *n* (%)			0.694
None	41 (16.9%)	39 (16.0%)	
Migraine specific	165 (67.9%)	160 (65.8%)	
Non-migraine specific	37 (15.2%)	44 (18.1%)	
Prior preventive treatment attempts, *n* (%)			0.699
None	137 (56.4%)	128 (52.7%)	
1 failed in the past year	66 (27.2%)	70 (28.8%)	
≥2 failed in the past year	40 (16.5%)	45 (18.5%)	
NRS scores (median ± IQR)	6 (4, 8)	7 (5, 10)	0.698
Number of comorbidities	2.27 ± 1.53	2.60 ± 1.55	0.166

SCG, superior cervical ganglion; BMI, body mass index; SD, standard deviation; NRS, numeric rating scale; IQR, interquartile range.

As shown in Table 2, the rate of pain relief at 24 h post-procedure was higher in the SCG cohort compared to the matched control cohort (*p* < 0.001). In addition, a higher percentage of subjects in the SCG cohort had freedom from pain within 24 h compared with those in the control cohort (*p* < 0.001). Superiority was met, as the 95% confidence interval (CI) of the mean difference fell within the predefined superiority margin of 15%. At 2 h following treatment, a difference in the rate of pain relief was also observed between the SCG cohort and control cohort, which reached statistical significance (*p* < 0.001). Overall, the SCG cohort had better freedom from headache within 24 h outcomes, whereas the control cohort was associated with worse outcomes (*p* < 0.001). Similarly, a significantly lower incidence of moderate to severe disability at 1 month during the follow-up period was reported in the SCG cohort as compared to the matched control cohort (*p* < 0.001). At the 1-month follow-up, a significant reduction in mean MMDs was observed in both cohorts compared to their baseline values. Furthermore, the change was statistically significant in the SCG cohort compared to the control group (*p* < 0.001). Although the quantifiable changes in MSQ scores at the 1-month follow-up visit were significantly higher than their baseline value in both cohorts, there was also a significantly greater improvement in MSQ score in the SCG block plus triptan group compared with the triptan alone group at 1 month (*p* = 0.036).

**Table 2:** Primary and secondary efficacy outcomes comparisons in the SCG and matched control cohorts.

Outcome		SCG cohort	Control cohort	Difference in rate	Rate ratio	*χ*^2^/*t* value	*P*-value
		(*n* = 243)	(*n* = 243)	(95% CI)	(95% CI)		
Pain relief, *n* (%)							
2 h headache relief		220 (90.5%)	179 (73.7%)	16.9% (10.2%–23.5%)	3.420 (2.042–5.728)	23.535	<0.001
2 h freedom from pain		169 (69.5%)	116 (47.7%)	21.8% (13.3%–30.4%)	2.500 (1.725–3.625)	23.831	<0.001
24 h sustained headache relief		178 (73.3%)	120 (49.4%)	23.9% (15.5%–29.0%)	2.807 (1.921–4.102)	29.182	<0.001
24 h sustained freedom from pain		156 (64.2%)	91 (37.4%)	26.7% (18.2%–31.3%)	2.995 (2.071–4.332)	34.783	<0.001
Monthly migraine days (mean ± SD)							
Baseline value		10.97 ± 2.95	9.07 ± 4.80			1.028	0.318
1-month post-treatment		3.33 ± 1.44	5.65 ± 1.75			−8.061	<0.001
MIDAS score, *n* (%)							
Baseline value	I	28 (11.5%)	34 (14.0%)			4.256	0.235
	II	37 (15.2%)	51 (21.0%)				
	III	69 (28.4%)	57 (23.5%)				
	IV	109 (44.9%)	101 (41.6%)				
1-month post-treatment	I	174 (71.6%)	114 (47.0%)			42.847	<0.001
	II	45 (18.5%)	49 (20.4%)				
	III	10 (4.1%)	35 (14.3%)				
	IV	14 (5.8%)	45 (18.3%)				
MSQ scores (mean ± SD)							
Baseline value		47.65 ± 4.64	47.33 ± 5.27			0.204	0.839
1-month post-treatment		78.54 ± 7.10	68.52 ± 11.35			2.275	0.036

SCG, superior cervical ganglion; SD, standard deviation; MIDAS, Migraine Disability Assessment; MSQ, Migraine-Specific Quality of Life questionnaire.

**Figure 3:**
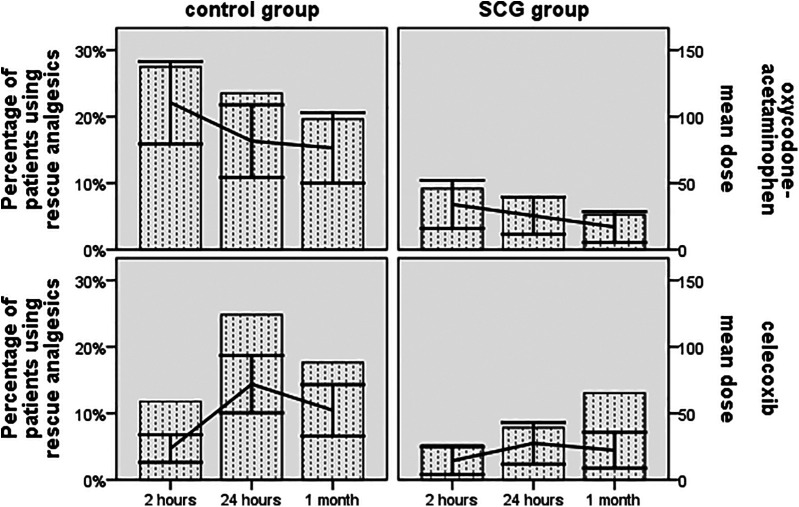
The proportion of subjects using rescue analgesics and the mean dose changes in rescue analgesics based on the World Health Organization analgesic ladder across all time points during the follow-up period.

The rate of subjects using rescue analgesia at 2 h was 5.2% and 9.2% for celecoxib and oxycodone-acetaminophen in the SCG cohort, respectively, as opposed to 11.8% and 27.5% in the control cohort, respectively (*p* = 0.063 and *p* < 0.001, respectively)(Figure 3). At the 24 h follow-up, 7.8% and 7.8% of subjects required the aforementioned analgesics for headache relief in the SCG cohort, respectively, while 24.8% and 23.5% were relieved with these analgesics in the control cohort, respectively (both *p* < 0.001). The rates of cases needing rescue celecoxib and oxycodone-acetaminophen at the 1-month follow-up were 7.2% and 5.2% for the SCG block-triptan-treated cases, respectively, vs. 17.6% and 19.6% for triptan-treated cases, respectively (*p* = 0.009 and *p* < 0.001, respectively).

No serious adverse events associated with US-guided SCG block, such as high-level subarachnoid or epidural block, nerve injury, vascular injection, visible hematoma, local anesthetic intoxication, allergic reaction, or arrhythmia, were observed. Furthermore, 5.1% of the subjects in the SCG cohort developed minor side effects associated with steroid and local anesthetics injection, including nausea, vomiting, and facial flushing; however, all of these symptoms resolved within 30 min. Regarding oral triptan-related side effects, 4.2% of cases experienced dizziness and somnolence in the SCG cohort, as did 5.0% of cases in the control cohort (*p* = 0.772).

## Discussion

Our retrospective analyses of the data indicated the superior therapeutic effectiveness of US-guided SCG block in conjunction with oral triptan for the management of acute migraine compared to triptan alone.

According to previous research, there were no changes in vessel caliber; however, a significantly increased cerebral blood flow was detected during the confirmatory angiography after the block of the SCG using local anesthetics (22). Moreover, an SCG block had the advantage of avoiding sympathectomy of the arm and cardiopulmonary tree by targeting the blockade to the uppermost part of the cervical sympathetic chain (23). As expected, there is growing evidence indicating that a block of the SCG can be employed in cases of trigeminal neuralgia, post-herpetic neuralgia, chronic headache, or facial pain (24). Consistent with our hypothesis, an adjunctive SCG block yielded significantly higher rates of pain relief and freedom from pain at 2 h, which persisted for 24 h after treatment compared to triptan alone. In accordance with our findings, Maeda et al. reported similar success in pain reduction for the treatment of headaches and orofacial pain, with maximum NRS pain scores significantly decreasing from 7.0 ± 0.7–4.5 ± 0.7 over 3 months after the application of an SCG block under US guidance (*p* = 0.014) (10). A previous open-label study reported that the incidence of migraine attacks decreased to one to six times per month, with freedom from attacks lasting 1/2–3/4 of a year, and extending to 1 year or more in most cases if repeated treatment with six to eight SCG blocks was performed (25). Our results also showed that the combination treatment of SCG block with triptan was superior to triptan alone, as evidenced by the significant reduction in the number of monthly migraine days at 1-month post-block (3.33 ± 1.44 vs. 5.65 ± 1.75 days).

Our data showed a greater improvement in headache-related disability in the SCG cohort, which exhibited a significantly lower proportion of individuals with disability (MIDAS scores ≥6) at 1 month after the procedure compared to the control cohort. This result aligns with a previous study that investigated the therapeutic role of cervical sympathetic nerve block in managing migraine attacks, which also reported a MIDAS score of >6 at 4 months after the block (26). Furthermore, the SCG cohort showed a greater improvement in MSQ scores, with a significant difference observed between the two cohorts when comparing the 1-month post-treatment values. According to a prior randomized study, the Headache Impact Test-6 scores of subjects who received repetitive sympathetic nerve blockades for chronic migraine were significantly lower than the placebo group at both 1 and 6 months post-block (25). When analyzing the usage of rescue analgesics, it is notable that there was a statistically significant difference between the sympathetic nerve block and control or placebo groups in previous studies on migraine (10, 25, 27). Our data showed that a significant decrease in the rate of using rescue analgesics, along with lower dosages, was observed in the SCG cohort at 2 h, 24 h, and 1 month post-treatment compared with the control cohort, thereby corroborating previous studies. A few case reports have shown that the US-guided technique with color Doppler makes SCG block a safe procedure (28). In the present study, only minor adverse events related to the US-guided SCG block procedure were found in 9.3% of cases, all of which were temporary, proving the safety of this technique.

There were several limitations. First, retrospective data may result in selection bias, information bias, or confounding. Second, the study had a single-center design, which may limit its applicability to other centers. Third, data collection was not conducted on pain-free days. Fourth, although the procedure is relatively safe and feasible, it is an invasive procedure and is highly dependent on the operator's experience. Further research should consider large samples from a well-designed, randomized, controlled study to verify our findings.

In conclusion, treatment comprising a US-guided SCG block in combination with triptans holds promise as an effective and safe strategy to relieve migraine attacks. Future studies are needed to make further comparisons with other commonly used techniques to incorporate this modality into routine clinical practice.

## Data Availability

The raw data supporting the conclusions of this article will be made available by the authors, without undue reservation.
