# Supplementary material for: Ultrasound-guided block of the superior cervical ganglion for migraine attacks: a propensity score-matched retrospective study
Source: Front Pain Res (Lausanne). 2025 Aug 28;6:1556654. doi: 10.3389/fpain.2025.1556654 (PMC12422891; doi:10.3389/fpain.2025.1556654)
Supplement: Supplementary file 1 [file Table1.docx]

**Supplemental Table 1 Migraine Disability Assessment Test.**

| **Items** | **Response** | |
| --- | --- | --- |
| On how many days in the last 3 months did you miss work or school because of your headaches? |  |  |
| How many days in the last 3 months was your productivity at work or school reduced by half or more because of your headaches? (Do not include days you counted in question 1 where you missed work or school.) |  |  |
| On how many days in the last 3 months did you not do household work (such as housework, home repairs and maintenance, shopping, caring for children and relatives) because of your headaches? |  |  |
| How many days in the last 3 months was your productivity in household work reduced by hall of more because of your headaches? (Do not include days you counted in question 3 where you did not do household work.) |  |  |
| On how many days in the last 3 months did you miss family, social or leisure activities because of your headaches? |  |  |
| What your Physician will need to know about your headache: |  |  |
| On how many days in the last 3 months did you have a headache? (If a headache lasted more than 1 day, count each day.) |  |  |
| On a scale of 0-10, on average how painful were these headaches? (where 0-no pain at all, and 10-pain as bad as it can be.) |  |  |
| **Total Score** |  | |

1. A total score is categorized into the following four disability grades:

0-5: minimal or infrequent disability; 6-10: mild disability; 11-20: moderate disability; ≥21: severe disability.

**Supplemental Table 2 Specific-for-IBD Nutritional Screening Tool.**

| **Saskatchewan Inflammatory Bowel Disease-Nutrition Risk Tool**  **(SaskIBD-NR Tool)** | | | |
| --- | --- | --- | --- |
| **Symptoms of Nause, Vomiting, Diarrhea or Poor Appetite for >2 weeks** | | | **Score** |
|  | 0 | No symptoms. |  |
|  | 1 | 1-2 symptoms. |  |
|  | 2 | ≥3 symptoms. |  |
| **Weight Loss (Unintentional)** | | | **Score** |
|  | 0 | No. |  |
|  | 1 | Unsure. |  |
|  | 0 | Yes: ＜5 lbs. |  |
|  | 1 | Yes: 5-10 lbs. |  |
|  | 2 | Yes: 10-15 lbs. |  |
|  | 3 | Yes: ＞15 lbs. |  |
| **Decreased Appetite** | | | **Score** |
|  | 0 | No. |  |
|  | 2 | Yes. |  |
| **Food Restriction** | | |  |
|  | 0 | No. |  |
|  | 2 | Yes. |  |
| **Total Score** | | |  |

1. Total Points:

0-2 points: Low Risk;

3-4 points: Medium Risk;

≥5 points: High Risk.

1. IBD= inflammatory bowel disease.

Differences between both scores:

1. The NRS-2002 tool primarily relied on weight loss and disease severity, it appeared insufficient on its own based on the poorer outcomes of patients not identified at-risk, indicating the sole use risks overlooking some patients who need and could benefit from early nutritional intervention.
2. The specific SaskIBD-NR tool included questions regarding gastrointestinal symptoms, the consumption of nutrients and weight loss, reflecting the activity or remission of IBDs, potential micronutrient deficiencies and protein energy malnutrition status, respectively, all of which are well-defined risk factors for malnutrition in patients with IBDs.
