# Supplementary material for: Ultrasound-guided block of the superior cervical ganglion for migraine attacks: a propensity score-matched retrospective study
Source: Front Pain Res (Lausanne). 2025 Aug 28;6:1556654. doi: 10.3389/fpain.2025.1556654 (PMC12422891; doi:10.3389/fpain.2025.1556654)
Supplement: Supplementary file 2 [file Table2.docx]

**Supplemental Table 2 The** **migraine-specific quality of life questionnaire.**

| **Items** | **Response** | |
| --- | --- | --- |
| Migraine interfered with how dealt with family. |  |  |
| Migraine interfered with leisure activities. |  |  |
| Difficulty performing work or daily activities due to migraine. |  |  |
| Kept from getting much done at work or home due to migraine. |  |  |
| Migraine limited ability to concentrate at work or for an activity. |  |  |
| Migraine limited the days felt energetic. |  |  |
| Skipped work or activity due to migraine. |  |  |
| Often needed help in handling routine tasks. |  |  |
| Stopped work or activity due to migraine. |  |  |
| Not gone to social activity due to migraine. |  |  |
| Felt frustrated due to migraine. |  |  |
| Felt like a burden due to migraine. |  |  |
| Afraid to disappoint others due to migraine. |  |  |
| **Total Score** |  | |

Participants responded to the items using a 6-point scale: “none of the time,” “a little bit of the time,” “some of the time,” “a good bit of the time,” “most of the time,” and “all of the time,” which are assigned scores of 1–6, respectively. Raw dimension scores were computed as a sum of item responses and rescaled from a 0–100 scale.

**Supplemental Table 2 Specific-for-IBD Nutritional Screening Tool.**

| **Saskatchewan Inflammatory Bowel Disease-Nutrition Risk Tool**  **(SaskIBD-NR Tool)** | | | |
| --- | --- | --- | --- |
| **Symptoms of Nause, Vomiting, Diarrhea or Poor Appetite for >2 weeks** | | | **Score** |
|  | 0 | No symptoms. |  |
|  | 1 | 1-2 symptoms. |  |
|  | 2 | ≥3 symptoms. |  |
| **Weight Loss (Unintentional)** | | | **Score** |
|  | 0 | No. |  |
|  | 1 | Unsure. |  |
|  | 0 | Yes: ＜5 lbs. |  |
|  | 1 | Yes: 5-10 lbs. |  |
|  | 2 | Yes: 10-15 lbs. |  |
|  | 3 | Yes: ＞15 lbs. |  |
| **Decreased Appetite** | | | **Score** |
|  | 0 | No. |  |
|  | 2 | Yes. |  |
| **Food Restriction** | | |  |
|  | 0 | No. |  |
|  | 2 | Yes. |  |
| **Total Score** | | |  |

1. Total Points:

0-2 points: Low Risk;

3-4 points: Medium Risk;

≥5 points: High Risk.

1. IBD= inflammatory bowel disease.

Differences between both scores:

1. The NRS-2002 tool primarily relied on weight loss and disease severity, it appeared insufficient on its own based on the poorer outcomes of patients not identified at-risk, indicating the sole use risks overlooking some patients who need and could benefit from early nutritional intervention.
2. The specific SaskIBD-NR tool included questions regarding gastrointestinal symptoms, the consumption of nutrients and weight loss, reflecting the activity or remission of IBDs, potential micronutrient deficiencies and protein energy malnutrition status, respectively, all of which are well-defined risk factors for malnutrition in patients with IBDs.
